# Hypertension, retinopathy, and acute kidney injury in dogs: A prospective study

**DOI:** 10.1111/jvim.15839

**Published:** 2020-07-17

**Authors:** Laura Pearl Cole, Rosanne Jepson, Charlotte Dawson, Karen Humm

**Affiliations:** ^1^ Clinical Science & Services, Royal Veterinary College Hatfield United Kingdom

**Keywords:** azotemia, fluid overload, hypertensive retinopathy, proteinuria

## Abstract

**Background:**

Systemic hypertension (SH) is a potential complication of acute kidney injury (AKI) in dogs.

**Objective:**

To describe the prevalence of SH and hypertensive retinopathy in dogs with AKI, to investigate the relationship between SH and severity of AKI and to assess possible factors associated with SH.

**Animals:**

Fifty‐two dogs with AKI.

**Methods:**

Prospective observational study of dogs presenting to a tertiary referral center that fulfilled the International Renal Interest Society (IRIS) guidelines for the diagnosis of AKI. Systolic blood pressure measurement, urine protein/creatinine ratio (UPCR), urine output, presence of hypertensive retinopathy and fluid overload (FO), survival to discharge and duration of hospitalization were subsequently assessed. The prevalence of SH was calculated and the relationship between SH and recorded factors was examined by nonparametric statistics.

**Results:**

The prevalence of SH (≥160 mm Hg) on admission or during hospitalization was 75% (39/52) and in 56% (22/39) of cases this was severe (≥180 mm Hg). Sixteen percent (7/43) of dogs had evidence of hypertensive retinopathy and 77% (24/31) dogs had UPCR >0.5. Forty‐two percent (22/52) dogs had FO on admission or during hospitalization. There was no association between SH and IRIS AKI grade, oligo/anuria, survival to discharge, duration of hospitalization or proteinuria. Dogs with FO on presentation were more likely to be hypertensive at admission compared to dogs without FO (*P* = .02). Dogs that did not survive to discharge were more likely to have FO (*P* = .007).

**Conclusions and Clinical Importance:**

Systemic hypertension is common in dogs with AKI. Systemic hypertension might be associated with FO, which itself is associated with nonsurvival. Monitoring for SH and FO is therefore warranted in dogs with AKI.

**Abbreviations**
ACVIM: American College of Veterinary Internal Medicine
AKI: acute kidney injury
CKD: chronic kidney disease
CRRT: continuous renal replacement therapy
FO: fluid overload
GFR: glomerular filtration rate
IRIS: International Renal Interest Society
SBP: systolic blood pressure
SH: systemic hypertension
TOD: target organ damage
UPCR: urine protein/creatinine ratio

## 1 INTRODUCTION

Acute kidney injury (AKI) is defined as an acute and abrupt decrease in kidney function resulting in abnormal glomerular filtration rate (GFR), tubular function and urine production.^1^ Systemic hypertension (SH) is a potential complication of renal injury with variable occurrence in both dogs and cats with chronic kidney disease (CKD) and AKI.^2^, ^3^, ^4^, ^5^

Systemic hypertension (SH) can lead to target organ damage (TOD), which includes hypertensive retinopathy, hypertensive encephalopathy, left ventricular hypertrophy, and progression of kidney disease. Hypertensive retinopathy occurs in both cats and dogs with CKD and hypertensive retinopathy and left ventricular hypertrophy occurs in dogs with glomerular disease secondary to leishmaniasis.^3^, ^6^, ^7^ The cause or causes of SH in kidney disease have yet to be fully elucidated but theories include impaired excretion of sodium and subsequent volume overload, excessive activation of the renin‐angiotensin‐aldosterone system, stimulation of the sympathetic nervous system via activation of chemosensitive afferent fibers and increase in systemic vascular resistance secondary to endothelial dysfunction.^8^, ^9^

In dogs with CKD, there is a positive correlation between systolic blood pressure (SBP) and degree of proteinuria and both SH and proteinuria are associated with disease progression and reduced survival time.^3^, ^10^ The prevalence of SH in CKD in various studies ranges between 9% and 93%.^11^ Based on retrospective studies 81% to 87% of dogs with AKI have SH.^4^ The literature on the effect of SH on the outcome of animals with AKI is sparse. Glomerular filtration rate is significantly reduced in hypertensive dogs compared to nonhypertensive dogs.^12^ However, in AKI in cats presence of SH has no effect on survival.^5^

The primary aims of this observational study were to describe the prevalence of SH and hypertensive retinopathy in dogs with AKI. The secondary aims were to investigate the relationship between SH and severity of AKI and to assess possible factors associated with SH. The hypotheses were that SH would be common in dogs with AKI, but ocular TOD would be less frequently detected. We further hypothesized that there would be no association between SH, severity of AKI or survival to discharge.

## 2 MATERIALS AND METHODS

The study was approved by the Royal Veterinary College Clinical Research Ethical Review Board (URN 2016 1590**).** Dogs presenting to a university referral teaching hospital diagnosed with community acquired AKI were prospectively recruited between July 2016 and November 2018. Dogs were eligible for inclusion if they fulfilled the International Renal Interest Society (IRIS) guidelines for the diagnosis of AKI; known access to nephrotoxins, serum creatinine increase >0.3 mg/dL over a 48 hour period or serum creatinine >1.6 mg/dL with 1 or more of the following criteria; evidence of renal tubular injury on urine analysis (renal glucosuria with normoglycemia, proteinuria with an inactive sediment, urinary casts, or both), imaging findings suggestive of AKI or oliguria (urine output <1 mL/kg/hr) over 6 hours.^13^

When available clinical records of the dogs were reviewed and dogs were excluded if there was any historical physical examination findings or previous clinicopathological data suggestive of CKD, including weight loss, polyuria, or both, and polydipsia greater than 4 weeks in duration, and previously documented azotemia with urine specific gravity <1.030. All dogs had renal ultrasound performed and dogs were excluded if diagnostic imaging findings were indicative of CKD. These included; small irregular kidneys and the presence of renal infarcts. Dogs were also excluded if they failed to have an initial SBP or serum creatinine concentration measurement, had a coexisting disease associated with SH (including hyperadrenocorticism, diabetes mellitus, pheochromocytoma), were on medication which could result in SH (glucocorticoids, ciclosporin, toceranib), or if they had been treated with antihypertensive drugs such as angiotensin‐converting enzyme inhibitors, angiotensin receptor blockers, beta blockers, calcium channel blockers, or acepromazine in the 48 hours before enrolment.

The grade of AKI was determined by the first serum creatinine concentration recorded from serum biochemistry (iLab 600, Instrumentation Laboratory, Cheshire, UK) when available, or bedside venous blood gas and metabolite sample (ABL90 FLEX, Radiometer UK Ltd, West Sussex, UK). The underlying cause of AKI was determined based on clinician interpretation of history and clinicopathological data including hematology (ADVIA 212Oi, Siemens Healthcare, Surrey, UK), serum biochemistry, microagglutination tests (Leptospirosis MAT, IDEXX Laboratories, Horsham, UK), infectious disease testing (SNAP 4Dx, IDEXX Laboratories, Horsham, UK), diagnostic imaging, urinalysis, urine protein/creatinine (UPCR), urine culture, and macroscopic and microscopic histopathological renal findings as appropriate.

Systolic blood pressure was measured within 12 hours of admission and at least once daily with Doppler sphygmomanometry. A standardized protocol adapted from the American College of Veterinary Internal Medicine (ACVIM) consensus guidelines was used; the dog was allowed to acclimatize for 5 to 10 minutes before placement of a cuff (with cuff width being 30%‐40% of the circumference of the limb at the cuff site) on either the forelimb or hindlimb, at the discretion of the operator. The first measurement was discarded and a total of 5 to 7 consistent values were taken, discarding any SBP reading >20% than the other SBP measurements. The average of these readings was recorded.^11^

Based on criteria in the ACVIM guidelines dogs were grouped based on their SBP and risk of TOD; normotensive (SBP < 140 mm Hg), prehypertensive (SBP 140‐159 mm Hg), hypertensive (SBP 160‐179 mm Hg) and severely hypertensive (SBP ≥180 mm Hg.^11^ Systemic hypertension was defined as a SBP≥160 mm Hg. Where individual dogs had more than 1 series of blood pressure readings performed on a given day, a median daily SBP was used for analysis. Antihypertensive therapy was at the discretion of the attending clinician.

Fundic examination was performed once within 48 hours of admission by an American or European specialist in Ophthalmology or a supervised resident in training to assess for presence of hypertensive retinopathy.^11^ Pupil dilatation was performed at the discretion of the ophthalmologist on a case‐by‐case basis. The ophthalmologists were unaware of the dog's SBP.

Urine output (UOP) was quantified either by measuring voided urine, weighing bedding, or urethral catheterization. Oliguria was defined as a urine output less than 1 mL/kg/hr for >6 hours.^2^ Fluid overload (FO) was diagnosed by the attending clinicians who determined this based on a daily dog assessment including monitoring for acute weight gain, serous nasal discharge, chemosis, subcutaneous edema or detection of cavitatory fluid with ultrasonography.^14^ Proteinuria was defined as UPCR >0.5 with an inactive sediment.^15^ Hypoalbuminemia was defined as serum albumin concentration < 2.6 g/dL.

The IRIS grade of AKI, serum albumin concentration and UPCR on admission were recorded. SBP, UOP and evidence of FO were reviewed daily. Any change in AKI grade or SH classification during hospitalization was recorded, alongside the use of antihypertensive agents and any extracorporeal therapy. Hospitalization duration, survival to discharge, survival at 3 months and follow‐up serum creatinine concentration and SBP where available were documented. The prevalence of SH and hypertensive retinopathy were calculated.

### 2.1 Statistical analysis

Data were assessed for normality by a Shapiro Wilks W test and a visual inspection of histograms. Normally distributed data were expressed as mean and standard deviation and non‐normally distributed data expressed as median and ranges. Statistical analysis was performed by statistical software (SPSS Statistics, Version 22.0, IBM).

Descriptive statistics were used to evaluate population characteristics. A Fisher's exact test was used to compare categorical data including number of dogs in each SBP category, AKI grade, presence of FO, proteinuria and hypoalbuminemia, and survival. A Bonferroni correction was used when comparing multiple categories. A Spearman's rank correlation coefficient was used assess for correlation between SBP, serum creatinine concentration and hospitalization duration. The level of statistical significance was set at *P* < .05 and adjusted as needed when using the Bonferroni correction.

## 3 RESULTS

Fifty‐six dogs presented with AKI between July 2016 and November 2018, of which 4 were excluded; 1 with prior history of antihypertensive therapy and 3 with incomplete data. Fifty‐two dogs were eligible for inclusion in the study. Of these dogs 49 were pure breeds; the most common pure breed was the Labrador (11/49) and 5/49 were sighthounds. Three out of 52 dogs were cross‐breeds. Nineteen (37%) were male neutered, 18/52 (35%) female neutered, 7/52 (14%) female entire and 8/52 (15%) male entire. The median age and mean weight of dogs were 57 months (range 3‐120) and 24.17 kg ±12.51, respectively. Dogs presented with a variety of causes of AKI (Table 1). Where a diagnosis was made, the most common were leptospirosis (12%, n = 6), prior nonsteroidal anti‐inflammatory drug use (12%, n = 6) and hypercalcemia (12%, n = 6).

**TABLE 1:** Causes of acute kidney injury in 52 dogs presenting to a tertiary referral center

Cause of AKI	Number of dogs
Leptospirosis	6
NSAID use – NSAID and GA – NSAID and pyelonephritis – NSAID alone	6 3 2 1
Hypercalcaemia – Hypercalcaemia – Hypercalcaemia and pyelonephritis	6 5 1
CRGV	4
Ischemia	3
Leishmania	2
Ethylene glycol toxicity	2
Grape and raisin toxicity	3
Glomerulonephritis	2
Unspecified toxin	1
GA	1
GA and rhabdomyolysis	1
Rhabdomyolysis	1
Diagnosis not made	14

Abbreviations: AKI, acute kidney injury; CRGV, cutaneous and renal glomerular vasculopathy; GA, general anesthesia; NSAID, nonsteroidal.

**FIGURE 1:**
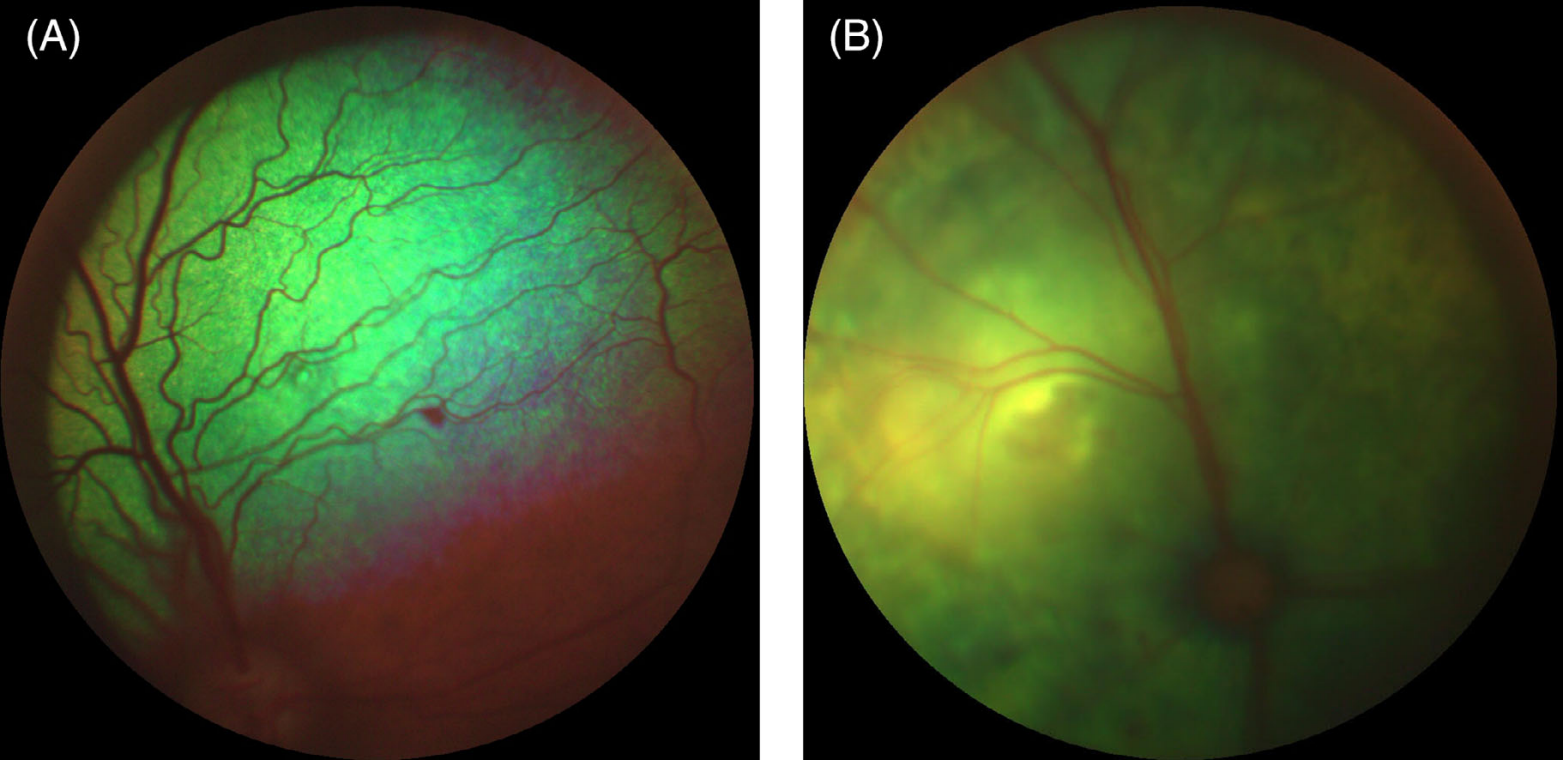
A, Focal retinal hemorrhage in a dog with acute kidney injury and systemic hypertension. B, Exudative retinal detachment in a dog with acute kidney injury and systemic hypertension

The median SBP on admission was 160 mm Hg (range 50‐275 mm Hg). The prevalence of SH (≥160 mm Hg) on admission was 54% (28/52) and in 43% cases (12/28) this was severe (≥180 mm Hg). The median peak SBP at any time point was 175 mm Hg (range 90‐300 mm Hg). The prevalence of SH at some point during hospitalization was 75% (39/52) which was severe in 56% cases (22/39) (Table 2). Forty‐three out of 52 dogs had a fundic examination during hospitalization and 21% (9/43) of these had evidence of ocular TOD. Two out of these 9 dogs, 1 in the normotensive category and the other in the prehypertensive, did not have a hypertensive reading at any point during their hospitalization. Fundic examination findings included retinal hemorrhage (9/9), retinal vessel tortuosity (2/9), exudative retinal detachment ‐complete (1/9)/multifocal (1/9) and hyphema (3/9) (Figure 1A,B). Thirty‐three percent (4/12) dogs that underwent a postmortem examination had evidence of left ventricular hypertrophy, 3 of 4 were hypertensive during hospitalization and 1 had retinal lesions.

**TABLE 2:** Prevalence of systemic hypertension at admission and during hospitalization in 52 dogs with acute kidney injury

	Blood pressure category	Prevalence
Admission	Normotensive (<140 mm Hg)	19% (10/52)
	Prehypertensive (140‐159 mm Hg)	27% (14/52)
	Hypertensive (160‐179 mm Hg)	31% (16/52)
	Severe hypertension (≥180 mm Hg)	23% (12/52)
During hospitalization	Normotensive (<140 mm Hg)	10% (5/52)
	Prehypertensive (140‐159 mm Hg)	15% (8/52)
	Hypertensive (160‐179 mm Hg)	33% (17/52)
	Severe hypertension (≥180 mm Hg)	42% (22/52)

Twenty‐five out of 39 (64%) dogs diagnosed with SH at some point during hospitalization were treated with antihypertensive drugs. Those hypertensive dogs that did not receive antihypertensive therapy had a short‐lived hypertension (<48 hours) or died shortly after hypertension was diagnosed. Of the treated dogs 22/25 (88%) received amlodipine alone, 1/25 (4%) received benazepril alone and 2/25 (8%) received multiple antihypertensive drugs. One dog received amlodipine, hydralazine and nitroprusside and the other received amlodipine, hydralazine and telmisartan. Both of these dogs had severe refractory hypertension (>200 mm Hg) and evidence of ocular TOD. The dogs given telmisartan and benazepril were categorized as AKI grade I and early grade III respectively. Choice of hypertensive therapy was based on the clinician's discretion. The median daily dose of amlodipine was 0.19 mg/kg (range 0.05‐0.45 mg/kg). This was given once a day in all dogs initially but was escalated to twice daily in 1 dog and given initially 3 times daily as incremental dose management in another in attempt to manage severe hypertension. Antihypertensive therapy was associated with a reduction in hypertension category in 14/25 (56%) of cases; 2/14 severe to normotensive, 7/14 severe to prehypertensive, 3/14 severe to hypertensive and 2/14 hypertensive to normotensive. Twelve of the 25 (48%) dogs given antihypertensive did not survive to discharge. Of the 13/25 (52%) dogs given antihypertensive therapy that did survive to discharge 7/13 (54%) were discharged on therapy, 4/13 (31%) had short lived hypertension in hospital and 2/13 (15%) were discharged to be euthanized.

The median serum creatinine concentration on presentation was 5.53 mg/dL (range 1.39‐19.56 mg/dL). Five dogs out of the 52 (10%) had grade I AKI, 2/52 (4%) grade II, 17/52 (33%) grade III, 17/52 (33%) grade IV and 11/52 (21%) grade V. Fourteen dogs (28%) had an increase in AKI grade during hospitalization. There was no correlation between initial SBP and serum creatinine on presentation (*r*_s_ = 0.182, *P* = 0.20). The prevalence of SH ranged between 50% and 91% across AKI grades (Table 3). There was no association between the presence of SH on admission or during hospitalization and IRIS AKI grade on presentation or increase in AKI grade during hospitalization.

**TABLE 3:** Prevalence of systemic hypertension and acute kidney injury grade on admission in 52 dogs with acute kidney injury

IRIS AKI grade	Prevalence of hypertension
I	60% (3/5)
II	50% (1/2)
III	94% (16/17)
IV	71% (12/17)
V	91% (10/11)

Abbreviations: AKI, acute kidney injury; IRIS, International Renal Interest Society.

Twenty‐nine out of 49 dogs were hypoalbuminemic (59%). The mean serum albumin concentration was 2.53 g/dL ±0.54 (n = 49). Thirty‐four dogs had an UPCR measurement, of which 1/34 had gross hematuria and 2/34 had an active sediment and so these dogs were not included in further analysis. The median UPCR in the 31 remaining dogs was 1.68 (range 0.1‐18). Twenty‐four out of 31 dogs (77%) had UPCR >0.5 of which 14/24 (58%) had a UPCR above 2. There was no association between presence elevated UPCR and hypoalbuminemia, or SH on admission or during hospitalization.

A urinary catheter was placed in 54% (28/52) dogs. When considering all methods of urine measurement the median urine output was 1.4 mL/kg/hr over the entire hospital period ranging from 0 to 25 mL/kg/hr. Forty‐two percent of dogs (22/52) were reported to be oliguric (urine output <1 mL/kg/hr for >6 hours), all of which received furosemide. Seven out of 20 dogs received a single bolus dose between 0.5 and 2 mg/kg, 2/20 dogs had a bolus between 0.5 and 1 mg/kg before a continuous infusion of 0.1 to 2 mg/kg/hr and 11/20 had a continuous infusion between 0.25 and 0.8 mg/kg/hr. In 2 dogs, the bolus dose of furosemide was not recorded. There was no association between the presence of SH and the presence of oliguria or anuria (*P* = .22). Six dogs had extracorporeal therapy, 4/52 (78%) had continuous renal replacement therapy (CRRT) and 2/52 (4%) had total plasma exchange. All 4 dogs that underwent CRRT were hypertensive on admission.

Twelve dogs (23%) were considered to have FO on admission and 10/52 (19%) developed FO during hospitalization. Forty‐two dogs (81%) received intravenous therapy before admission and 94% (49/52) received fluids at some point during hospitalization. In those dogs with FO on presentation, fluid therapy was administered after furosemide in 6/10 (60%), extracorporeal therapy in 2/10 (20%) and for replacement of varying insensible and sensible losses in 2/10 (20%) dogs. There was a significant association between FO on admission and SH on presentation (*P* = .02) but there was no association between SH and the development of FO in hospital.

Twenty‐five (48%) dogs survived to discharge and 3 dogs (6%) were discharged home for euthanasia. Of those dogs that did not survive to discharge 22/24 (92%) were euthanized and 2/24 (8%) died. The mean duration of hospitalization for the surviving dogs was 8.48 days ±4.13. There was no correlation between the SBP and hospitalization duration for dogs that survived (*r*s = 0.225, *P* = .28). There was no association between survival to discharge and SH on admission or during hospitalization, nor was there an association between IRIS AKI grade and survival. There was an association between the presence of FO at any point during hospitalization and survival to discharge (*P* = .007).

In those dogs that survived to discharge, 32.0% (8/25) were lost to follow‐up. Out of the remaining dogs 29% (5/17) died within 3 months. Of the 5 dogs that died within 3 months, 2/5 were euthanized directly as a result of their AKI, 2/5 were euthanized as result of an underlying neoplastic process and 1/5 died of unrelated causes. Overall 3‐month survival rate of all dogs available for follow‐up was 28% (12/44). The presence of SH at admission and during hospitalization was not associated with 3‐month survival. Five out of 10 (50%) dogs that had serum creatinine measured within the 3 month follow‐up period after discharge had a serum creatinine above the upper limit of the laboratory defined value of >1.63 mg/dL. All of these dogs were hypertensive at some point during hospitalization. Four out of 5 dogs that had blood pressure measurements between 2 and 12 week after discharge were found to be hypertensive and on therapy.

## 4 DISCUSSION

This prospective study shows that the prevalence of SH is high in dogs with community acquired AKI and appears to increase during hospitalization; 54% of dogs were hypertensive on presentation and 43% had severe hypertension. These values increased to 75% and 56% respectively during hospitalization. These results are similar to the currently reported SH occurrence rate of 81% to 87% in dogs with AKI during hospitaliszation.^4^ There is therefore a clear requirement for frequent blood pressure monitoring of animals hospitalized with AKI.

Hypertensive retinopathy was detected in 16% dogs in this study. This is less than reported in a study of all causes of hypertension, which report a prevalence of 62%.^16^ However, the prevalence is similar to that previously described in dogs with CKD.^3^ The low prevalence of hypertensive retinopathy could be explained by the low number of dogs presenting with severe hypertension, the low rate of repeat ophthalmological examinations, or failure to detect early retinal lesions, as pupil dilatation was not performed in all dogs. Two dogs, 1 in the normotensive category and 1 in the prehypertensive category, also had retinopathy consistent with hypertension. One of the dogs had AKI secondary to a combination of presumed renal hypotension secondary to general anesthesia and laparoscopy, and nonsteroidal anti‐inflammatory drug therapy. Retinal hemorrhage occurs in humans undergoing laparoscopic surgery suspected to be secondary to increased retinal venous pressure^17^ and therefore could explain this finding. Alternatively, these findings could be explained by underestimation of the diagnosis of hypertension as a result of inherent inaccuracies of indirect measures of SBP, or could suggest that macroscopic retinal vascular changes are not sensitive indicators of early ocular TOD. Studies of early stage untreated essential hypertension in humans failed to find a relationship between retinal microvascular changes and other validated markers of TOD such as 24‐hour ambulatory blood pressure monitoring, 24‐hour urine collection for microalbuminuria, echocardiography, and carotid ultrasonography.^18^ Despite the seemingly low prevalence of hypertensive retinopathy in AKI, systemic heparinization in the presence of retinal hemorrhage could lead to severe retinal damage. An ophthalmological exam should therefore be considered in all AKI dogs before CRRT.

There was no association between evidence of hypertensive retinopathy and severity of SH. This might be the result of a type II error because of the low numbers of dogs with evidence of hypertensive retinopathy or the temporal relationship between detection of SH and fundic examination. Fundic examination was standardized to occur in the first 48 hours and the number of severely hypertensive dogs at admission was low. Another factor shown to affect the development of TOD in people is the variability of SBP; greater variability of SBP has been shown to associated with greater risk of TOD.^19^ SBP variability was not assessed in this study but is something that should be considered in future studies.

Left ventricular hypertrophy, indicative of cardiac TOD has been reported in 91% of hypertensive dogs with leishmaniasis, of which over half of the dogs had kidney disease.^7^ In our study, 4/12 dogs that underwent a postmortem examination had evidence of left ventricular hypertrophy, of which 3 quarters of them were noted to be hypertensive during hospitalization and 1/4 had retinal changes. These findings suggest a degree of chronicity of the SH and therefore might indicate a failure to diagnose CKD in this subset of dogs. However, the possibility of the AKI occurring secondary to SH cannot be excluded. These results suggest other diagnostic tests such as echocardiography should be used alongside a fundic examination to further assess for TOD.

Proteinuria has been associated with SH and persistent proteinuria is considered a finding indicative of TOD.^11^, ^20^ Studies report a positive but nonlinear correlation between SBP and UPCR in dogs.^10^, ^12^, ^21^ Proteinuria might also be the result of primary glomerular disease. A UPCR >2 alongside hypoalbuminemia is considered consistent with glomerulonephropathy^15^ and SH has been shown to associated with glomerular disease in dogs.^7^ In our study, over 75% dogs had a UPCR >0.5 of which 58% had UPCR >2. Few previous studies have quantitatively assessed proteinuria in AKI dogs; 78% dogs with leptospirosis were reported to have an elevated UPCR^22^ and in another study of 125 dogs UPCR values between 0.09 and 72 were reported in dogs with AKI of all causes and the degree of proteinuria was significantly greater in nonsurvivors.^23^ In the current study there was no association between proteinuria and hypoalbuminemia, SH and survival to discharge. Failure to detect an association between SH and proteinuria might be the result of a type II error because of the small number of dogs with UPCR measurement or secondary to the effect of treatment; hypertensive dogs were treated at the clinician's discretion and many had improvement in their SBP category which could have masked an association between SH and proteinuria. Alternatively, the proteinuria could have been predominantly tubular in origin rather than a result of glomerular hypertension. Future studies with renal biomarkers of glomerular and tubular injury such as fractional excretion of IgG and IgM and urine neutrophil gelatinase‐associated lipocalin (NGAL) alongside trending of UPCR in dogs with AKI over time might help determine the origin of this proteinuria and could have consequences of the choice of antihypertensive therapy.

There was no association between the presence of SH and IRIS AKI grade or absolute serum creatinine concentration. This is similar to findings in cats with AKI and in dogs with cardiorenal syndrome.^5^, ^24^ These finding suggests SH can occur at all grades of AKI. Interestingly, canine studies have shown a reduction in GFR correlates with an increase in SBP.^10^, ^12^ In an experimental canine study, hypertensive dogs had a significantly reduced GFR and increase in renal tubular lesions and fibrosis compared to normotensive dogs in the weeks to months after AKI, suggesting SH has appreciable adverse effect on kidney structure and function^12^ In the current study SH was treated at the clinician's discretion and hypertensive category reduced in around half of the dogs. This intervention could have masked the potential effect of SH on disease progression and outcome. Furthermore, the number of cases loss to follow‐up was high and up sampling was not standardized reducing the study's power to detect the long‐term effect of SH on kidney function. Finally, the IRIS guidelines used in this study for grading AKI are based on serum creatinine concentrations alone which is not considered an accurate indicator of GFR.^13^, ^25^ Future studies should therefore consider the routine use of commercial measures of GFR at standardized follow‐up time points.

There was no association between SH at admission or development during hospitalization and survival nor between AKI grade and survival to discharge. The failure to detect an association between SH and survival to discharge is similar to that reported in cats with AKI.^5^ Dogs enrolled had no pretreatment with antihypertensive agents and therefore we can reliably assess the effect of previously untreated SH on admission with disease severity and outcome. However, during hospitalization antihypertensive therapy was administered at the clinician's discretion and in around half of treated dogs their hypertension resolved. It is therefore difficult to elucidate the effect of persistent hypertension on outcome in this study. It is plausible that antihypertensive therapy masked the effect of SH on survival. The lack of association between severity of AKI and outcome is surprising and contrary to previous studies of AKI whereby increased AKI grade was associated with worsened outcome.^23^, ^26^, ^27^ This difference could be explained by differences in causes of AKI in our population versus others and the over‐reaching effect of etiology on outcome in AKI irrespective of absolute serum creatinine concentration. The study's small size, particularly the number of dogs in lower AKI grades, might also contribute to failure to detect an effect of AKI grade and outcome.

Of those dogs which survived and for which information was available, 50% were azotemic on 1 or more occasion after discharge. All of these dogs were hypertensive at some point during hospitalization. Inferences on the effect of SH on development of CKD cannot be made given the small number of dogs, but this supports the need for regular monitoring of dogs after an episode of AKI. Only 5 dogs had follow‐up blood pressure, of which 4/5 were persistently hypertensive and on antihypertensive therapy. Monitoring blood pressure after discharge is particularly important considering human AKI dogs are more likely to develop hypertension during follow‐up.^28^

Despite the frequent occurrence of SH in dogs with kidney disease the pathogenesis is poorly understood. Suggested etiologies include volume impaired excretion of sodium leading to volume overload, excessive activation of the renin angiotensin‐aldosterone system, stimulation of the sympathetic nervous system secondary to activation of chemosensitive afferent neurons by local ischemia and inflammation, reduced bioavailability of the vasodilator nitric oxide and increased production of the vasoconstrictor endothelin. ^8^, ^9^ In this study the presence of SH at admission was associated with the presence of FO at admission suggesting a role for volume and sodium excess in the pathogenesis of hypertension in this population. It is unclear why this relationship did not persist during hospitalization but the authors hypothesize it is related to the duration of FO; during hospitalization daily frequent monitoring of weight encourages early detection of FO. This might prompt the de‐escalation of fluid therapy before the development of clinically detectable volume overload. In human dialysis, dogs' volume status, particularly those with FO are associated with both predialysis and postdialysis blood pressure. Blood pressure falls during hemodialysis with fluid removal and the decrease in blood pressure is greatest with larger amounts of fluid removal and with higher ultrafiltration rates.^29^ In the current study, 4 dogs underwent CRRT as part of their management, all of which were hypertensive. SH did not resolve after therapy in any of the dogs, despite resolving their FO, suggesting this might only partly contribute to SH in AKI. Further studies of volume status in dogs with AKI and SH, using standardized measures of volume status such as bioimpedance are required. In this study the presence of FO at any point during hospitalization was also associated with a reduced survival to discharge and this is consistent with studies in critical dogs and people undergoing renal replacement therapy.^30^, ^31^ This finding highlights the need for careful monitoring and judicious fluid therapy dogs with AKI.

Amlodipine at a daily dose range of 0.1 to 1.2 mg/kg was the predominant therapy for hypertension used in this study. One dog received a dose above the recommended range of 0.1 to 0.5 mg/kg PO^11^ similar to a previous study where doses up to 1 mg/kg were required.^4^ Despite treatment, the hypertensive category only reduced in around half of the treated dogs. Failure to achieve control is likely because of failing to appropriately adjust antihypertensive medication in light of persistent hypertension, particularly in those dogs that had shorter survival times. Additional contributing factors include; the potential for reduced oral bioavailability of amlodipine in critically ill dogs^32^ and secondary glomerular hypertension as a consequence of preferential afferent arteriole vasodilatation with amlodipine therapy.^11^ Further studies assessing amlodipine levels, proteinuria, and continuous blood pressure measurements in critical AKI dogs are required to best determine the dose and role of the drug in these dogs.

This study had several limitations. It was conducted in a referral center and therefore the availability of complete medical records before referral was variable and the majority of dogs received treatment, including fluid therapy, before referral. The diagnosis of FO involved subjective assessment and was therefore less accurate than determining fluid balance based on calculations of fluid in and fluids out and the use of bioimpedance techniques.

AKI grade was determined based on the first recorded serum creatinine concentration and therefore the method of measurement was not standardized. This could lead to inter‐individual variation in serum creatinine concentration and could affect subsequent AKI grading. Furthermore, only 7 dogs with IRIS AKI grade I and II were enrolled reducing the distribution of data. Being a clinical study there was no predefined guidelines for management of SH, nor was there a control untreated group of dogs. This, therefore, limits the study's power to determine the effect of SH on AKI severity and outcome. Furthermore, there was no standardization of management of dogs once discharged and there was a high occurrence of loss to follow‐up of dogs that survived to discharge. This, thereby, limits the assessment of the effect of SH on long‐term kidney function. Further studies should focus on the efficacy of predefined antihypertensive therapy and routine follow‐up including serum creatinine and other biomarkers of kidney function, urinalysis and GFR measurements to fully assess the effect of SH and development of CKD.

Situational hypertension, an increase in blood pressure as a result of adrenergic stimulation during situations of stress or anxiety, is difficult to control in any clinical study.^11^ Situational hypertension has been documented in hospitalized dogs, in particular greyhounds.^31^, ^33^, ^34^ Furthermore, greyhounds have significantly higher SBP than other breeds.^35^ In this study, there were 5 sighthounds and 3/5 were considered hypertensive, of which 2 had SBP over 180 mm Hg. Although in the majority of studies the increase in blood pressure in the hospital was marginal, values up to 200 mm Hg are reported and therefore the presence of situational hypertension could lead to misclassification of dogs in all hypertensive categories.^35^, ^36^ Despite hospital protocols being in place to minimize the effect of stress on the blood pressure readings and following ACVIM guidelines^11^ to measure blood pressure, the prevalence of SH in this study might have been increased by dogs with solely situational hypertension. Continuous blood pressure monitoring would be required to fully mediate the effect of situational hypertension.

Doppler sphygmomanometery is a recognized indirect measure of blood pressure. However, indirect blood pressure measurements have been shown to underestimate SBP in the hypertensive dog and therefore using this methodology might have underestimated the true prevalence of SH.^37^ The gold standard technique for measurement of blood pressure is direct arterial catheterization, but is not practical in this subset of dogs. Telemetric blood pressure measurement could be useful both during hospitalization and after discharge to reliably detect SH, TOD and the effect of treatment in dogs with AKI.^38^

In summary, SH is common in dogs with AKI. However, hypertensive retinopathy appears to be uncommon. Systemic hypertension can occur at all grades of AKI and therefore SBP should be monitored in all dogs with AKI irrespective of severity. Systemic hypertension on admission does not appear to affect outcome, however, in light of the routine use of antihypertensives the effect of persistent SH on outcome cannot be fully elucidated from this study. The relationship between FO and SH needs to be further explored but considering FO was associated with worsened outcome measures should be taken to monitor for, and prevent FO in dogs with AKI.

## CONFLICT OF INTEREST DECLARATION

Authors declare no conflict of interest.

## OFF‐LABEL ANTIMICROBIAL DECLARATION

Authors declare no off‐label use of antimicrobials.

## INSTITUTIONAL ANIMAL CARE AND USE COMMITTEE (IACUC) OR OTHER APPROVAL DECLARATION

Authors declare no IACUC or other approval was needed.

## HUMAN ETHICS APPROVAL DECLARATION

Authors declare human ethics approval was not needed for this study.
